# Sitagliptin on Carotid Intima-Media Thickness in Type 2 Diabetes Mellitus Patients and Anemia: A Subgroup Analysis of the PROLOGUE Study

**DOI:** 10.1155/2020/8143835

**Published:** 2020-05-11

**Authors:** Zhengri Lu, Genshan Ma, Lijuan Chen

**Affiliations:** ^1^School of Medicine, Southeast University, Nanjing, China; ^2^Department of Cardiology, Zhongda Hospital, Southeast University, Nanjing, China

## Abstract

**Introduction:**

Randomized clinical trials have not shown an additional clinical benefit of sitagliptin treatment over conventional treatment alone. However, studies of sitagliptin treatment have not examined the relationship between anemia and treatment group outcomes.

**Methods:**

The PROLOGUE study is a prospective clinical trial of 442 participants with type 2 diabetes mellitus (T2DM) randomized to sitagliptin treatment or conventional treatment which showed no treatment differences [Estimated mean (± standard error) common carotid intima-media thickness (CIMT) was 0.827 ± 0.007 mm and 0.837 ± 0.007 mm, respectively, with a mean difference of -0.009 mm (97.2% CI −0.028 to 0.011, *p* = 0.309) at 24 mo of follow-up]. This is a post hoc subanalysis using data obtained from the PROLOGUE study; the study population was divided into anemic groups (*n* = 94) and nonanemic group (*n* = 343) based on hemoglobin level. And we analyzed for the changes in each CIMT parameter from baseline to 24 months in subgroups.

**Results:**

The treatment group difference in baseline-adjusted mean common carotid artery- (CCA-) IMT at 24 months was −0.003 mm (95% CI −0.022 to 0.015, *p* = 0.718) in the nonanemic subgroup and −0.007 mm (95% CI −0.043 to 0.030, *p* = 0.724) in the anemic subgroup. Although there were no significant differences in the other CIMT parameters between the treatment groups in the anemic subgroup, the changes in mean and max ICA-IMT at 24 months in the nonanemic subgroup were significantly lower in the sitagliptin group than the conventional group [−0.104 mm (95% CI −0.182 to −0.026), *p* = 0.009 and −0.142 mm (−0.252 to −0.033), *p* = 0.011, respectively].

**Conclusion:**

These data suggest that nonanemia may indicate a potentially large subgroup of those with T2DM patients that sitagliptin therapy has a better antiatherosclerotic effect than conventional therapy. Further research is needed to confirm these preliminary observations.

## 1. Introduction

Atherosclerosis is an inflammatory disease involving the interaction of genetic and environmental factors. It is usually caused by hypertension, hyperlipidemia, diabetes, smoking, and unhealthy diet, which is the leading cause of vascular disease globally. Among them, diabetes mellitus is not only a disorder of glucose metabolism but is also considered to be a high-risk disease that is causing atherosclerosis. A prospective cohort study has shown that the lifetime risk of vascular death in diabetic patients without previous coronary heart disease (CHD) is as high as the risk of CHD only [1]. Therefore, active and effective interventions are needed, including dietary change, physical exercise, and medication to reduce the prevalence of diabetes.

The carotid intima-media thickness (CIMT) is a surrogate marker of atherosclerosis, which is the combined thickness of the tunica intima and media of a circulatory vessel detectable noninvasively with ultrasonographic techniques [2]. On the one hand, CIMT is directly associated with the risk of myocardial infarction and stroke and is considered to be an effective tool for early diagnosis of atherosclerosis [3, 4]. Some studies suggest that the progression of carotid IMT in coronary artery disease (CAD) can be used to predict coronary events and related mortality [5–7]. The link between CIMT and CAD may be related to inflammation, which is recognized to play a critical role in the pathogenesis of atherosclerosis [8]. It has been recognized that the pathogenesis of increased CIMT and CAD are both related to atherosclerosis. These findings emphasize the importance of recognizing and managing the early stages of atherosclerosis for effective prevention of CAD. On the other hand, assessing the efficacy of drugs for diabetes is an active area of therapeutic research in metabolic diseases. Some studies have attempted to evaluate the effects of various drugs on CIMT changes. A systematic review demonstrated that statins can reduce CIMT by lipid decrease [9]. Another meta-analysis suggested that alpha-glucosidase inhibitors (alpha-GIs) can attenuate the CIMT progression in patients with impaired glucose tolerance or type 2 diabetes mellitus (T2DM) [10]. However, to date, there are less data on dipeptidyl peptidase-4 (DPP-4) inhibitors and glucagon-like peptide-1 (GLP-1) receptor agonists associated with CIMT progression. A meta-analysis of 5 studies revealed that there was no statistically significant decrease in IMT by GLP-1 based therapies [11]. DPP-4 inhibitors are a class of antihyperglycemic drugs that can effectively increase the concentration of insulin and control blood glucose levels. In addition, DPP-4 inhibitors may have additional effects beyond blood glucose control, such as antiatherosclerotic effects [12, 13]. Several researches using animal models have confirmed that DPP-4 inhibitors significantly suppressed atherosclerotic lesions mainly through the actions of GLP-1 and glucose-dependent insulinotropic polypeptide (GIP) [14–18]. In addition, clinical studies have also demonstrated the anti-inflammatory and antiatherosclerotic effects of DPP-4 inhibitors [19, 20]. However, some large-scale clinical trials have found that the DPP-4 inhibitors neither increase nor decrease the incidence of cardiovascular events [21–23]. In addition, some studies have shown that DPP-4 inhibitors can reduce the CIMT increase [24, 25]. Sitagliptin (a DPP-4 inhibitor) and liraglutide (a GLP-1 receptor agonist) treatment improved arterial stiffness by reducing oxidative stress in T2DM patients [26, 27]. But the PROLOGUE trial did not find that sitagliptin showed an additional effect in inhibiting the progression of CIMT. Therefore, the antiatherosclerotic effect of DPP-4 inhibitors has not been fully elucidated.

Diabetes patients are often accompanied by anemia, which is associated with an increased risk of adverse cardiovascular events and kidney disease [28–30]. The prevalence of anemia in patients with diabetic nephropathy is higher than that with other types of chronic kidney disease (CKD), and the severity of anemia is increasing gradually with the deterioration of renal function. Hemoglobin concentration in patients with diabetes continued to decrease significantly even without nephropathy [31]. Although previous studies have reported abnormal hemoglobin concentrations in patients with diabetes [31, 32], other studies have also shown an association between anemia and cardiovascular disease (CVD) in patients with diabetes mellitus and in those with chronic kidney disease (CKD) [33, 34]. But the relationship between anemia and subclinical atherosclerosis markers in patients with T2DM remains somewhat elusive. In general, we rarely consider anemia as a factor in the development of atherosclerosis in patients with diabetes. Although the prospective randomized clinical trial of sitagliptin has not shown other clinical benefits, the effects of baseline anemia events on CIMT after sitagliptin treatment are unclear. We used data from the PROLOGUE study to test the hypothesis that certain patients in low- and high-risk subgroups were more likely to benefit from sitagliptin.

## 2. Methods

### 2.1. Study Design

This is a *post hoc* analysis using data from the PROLOGUE study, a multicenter, randomized, prospective, open-label, blinded endpoint trial carried out at 48 institutions in Japan that evaluated 442 patients with T2DM between June 2011 and September 2012 [35]. The inclusion criteria were age ≥ 30 years and presence of T2DM with HbA1c (JDS) 6.2–9.4% (JDS indicates Japan Diabetes Society value that is expressed as 0.4% lower than National Glycohemoglobin Standardization Program value) despite treatment with diet, exercise, and/or conventional antidiabetic agents (except incretin-related therapy) [36]. Patients with the administration of DPP-4 inhibitors and/or GLP-1 analogues before randomization and heart failure with New York Heart Association functional class III or IV were excluded. Study inclusion and exclusion criteria have been published previously. There were two treatment groups in PROLOGUE: sitagliptin treatment (“sitagliptin group”, *n* = 222) and conventional glucose-lowering treatment (“conventional group”, *n* = 220). The PROLOGUE study primary endpoint was the change in mean common carotid artery- (CCA-) IMT at 24 months after treatment randomization. Other CIMT parameters, including the internal carotid artery- (ICA-) IMT, were secondary endpoints. The study was approved by all participating institutional review boards, and all study participants gave informed consent. The full study protocol can be found in previously published research.

In the *post hoc* analysis, anemia was defined according to the concentration of hemoglobin, which is the standard index of anemia. According to the World Health Organization (WHO) definition of anemia: hemoglobin < 13 g/DL in men and <12 g/DL in women [37]. The study population was divided into the anemic group (*n* = 94) and the nonanemic group (*n* = 343) based on this definition. And we analyzed for the changes in each CIMT parameter from baseline to 24 months in subgroups.

### 2.2. Statistical Analysis

Normally distributed continuous data were shown as mean ± standard deviation and compared using a two-tailed unpaired Student's *t*-test. Categorical variables were expressed as frequencies (%) and compared using the Chi-squared test wherever appropriate. The baseline-adjusted means of each parameter were estimated by analysis of covariance with treatment effect and age, sex, statin use, prerandomization treatment type, baseline HbA1c, baseline office systolic blood pressure, baseline maximum IMT, and the baseline value of each parameter as covariates. A two-tailed *p* value < 0.05 was definitely statistically significant. Statistical analyses were performed with SPSS Statistics Software (version 25.0; IBM SPSS, Armonk, New York, United States of America).

## 3. Results

### 3.1. Baseline Characteristics of Study Subjects

**Figure 1:**
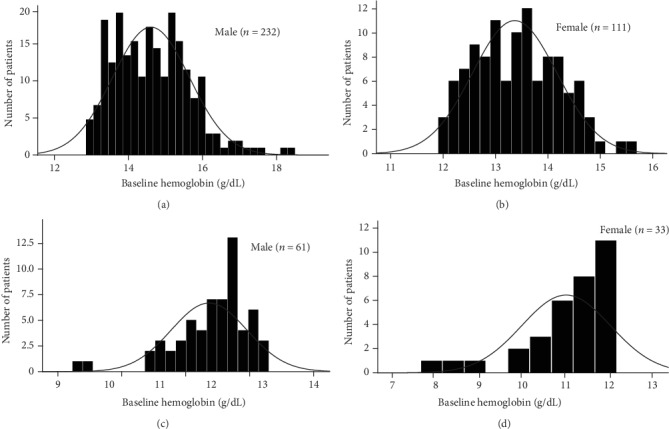
Distribution of the baseline hemoglobin concentrations across the entire cohort of male and female patients. (a) and (b) indicate the number of male and female nonanemic patients, respectively. While (c) and (d) indicate the number of male and female anemic patients, respectively.

The baseline demographic and clinical characteristics of the 442 study participants (222 subjects in the sitagliptin group and 220 in the conventional treatment group) have been previously reported. There were no significant differences in terms of the clinical parameters between the two groups. In this *post hoc* analysis, we compared the baseline demographics and clinical variables in subgroups (Table 1). As shown by the baseline hemoglobin distribution in Figure 1, the hemoglobin concentrations of male and female nonanemic patients were 14.642 ± 1.030 g/DL and 13.366 ± 0.807 g/DL, respectively. And the hemoglobin concentrations of male and female anemic patients were 11.944 ± 0.732 g/DL and 10.988 ± 1.018 g/DL, respectively. The baseline variables were similar between treatment groups, except more prevalent prior to chronic heart failure in patients treated with conventional in the anemic subgroup. The average body mass index, diastolic blood pressure, fasting plasma glucose, and prevalence of dyslipidemia were modestly higher in the nonanemic subgroup. Baseline HbA1c levels in the two subgroups were around 7.0%. No significant difference in CIMT parameters between the treatment groups in each subgroup.

### 3.2. Effect of Sitagliptin on Metabolic Factors and Carotid IMT in Subgroups

**Figure 2:**
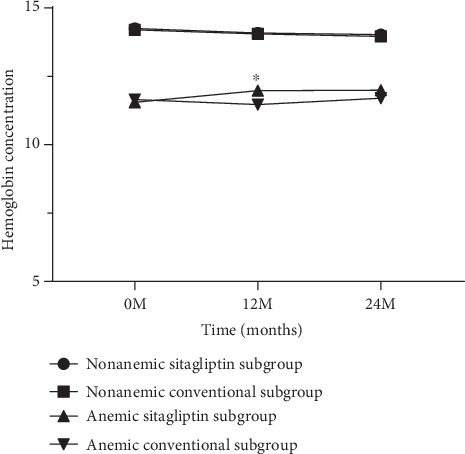
Time course of hemoglobin concentration of anemic and nonanemic subgroups during treatment. Graph shows sex-, age- and baseline-adjusted mean (±standard error) at 12 and 24 months. Hemoglobin at 12 months shows a significant difference in the anemic subgroup. ^∗^*p* = 0.027 vs. conventional subgroup.

The effects of sitagliptin on metabolic factors and CIMT have been previously reported. In conclusion, sitagliptin treatment has a more effective hypoglycemic effect than conventional treatment. In this *post hoc* analysis of the PROLOGUE study, we also found a similar result. The changes in HbA1c at 24 months in the nonanemic subgroup were significantly lower in the sitagliptin group than the conventional group [−0.146 mm (95% CI −0.282 to −0.010), *p* = 0.035]. However, regarding blood pressure, non-HDL cholesterol, serum creatinine, and estimated glomerular filtration rate (eGFR), there was no significance between treatment group differences in changes from baseline to 24 months. As shown in Figure 2, hemoglobin concentration was slightly higher in patients treated with sitagliptin than in patients treated with conventional in the anemic subgroup (11.981 ± 0.180 g/DL and 11.473 ± 0.178 g/DL, *p* = 0.027). The treatment group difference in baseline-adjusted mean CCA-IMT at 24 months was −0.003 mm (95% CI −0.022 to 0.015, *p* = 0.718) in the nonanemic subgroup and − 0.007 mm (95% CI −0.043 to 0.030, *p* = 0.724) in the anemic subgroup (Tables 2 and 3). Although there were no significant differences in the other CIMT parameters between the treatment groups in the anemic subgroup, the changes in mean and max ICA-IMT at 24 months in the nonanemic subgroup were significantly lower in the sitagliptin group than the conventional group [−0.104 mm (95% CI −0.182 to −0.026), *p* = 0.009 and −0.142 mm (−0.252 to −0.033), *p* = 0.011, respectively].

### 3.3. Effect of Sitagliptin on the Ultrasonic Tissue Characteristics of the Carotid Wall in Subgroups

There were no significant differences in the GSM-Plaque between the two treatment groups in each subgroup at baseline and 24 months (Tables 1–3).

### 3.4. Antidiabetic and Other Medications in Subgroups during the Study

There were no significant differences in the baseline frequency of noninvestigational drugs except glinide between the treatment groups in each subgroup. In each conventional group, the additional use of sulfonylureas, metformin, *α*-glucosidase inhibitor, and thiazolidinedione increased during the 24 mo observation period. However, with the exception of metformin, there was no increase in drug use in each sitagliptin group (Table 4).

## 4. Discussion

The present study, a subgroup analysis using data obtained from the PROLOGUE study showed that sitagliptin treatment significantly inhibited the progression of mean and max ICA-IMT after 24 months observation period in the nonanemic subgroup, while the conventional treatment did not affect the ICA-IMT. But in the anemic subgroup, there was no significant difference in changes with CIMT between sitagliptin and conventional treatment groups. Although there was no significant difference in CCA-IMT between the treatment group in each subgroup, this analysis shows that sitagliptin treatment more effectively inhibited the progression of CIMT than conventional treatment in nonanemic T2DM patients. However, patients with T2DM and anemic do not seem to benefit from sitagliptin treatment.

Although anemia is a common complication in diabetic patients, it is often overlooked. And the relationship between anemia and ICA-IMT in patients undergoing sitagliptin treatment has not been described. This may be an important issue. Anemia is a strong risk factor for death in people with diabetes due to CVD [32–34]. Although many previous clinical studies have shown that there is a correlation between hemoglobin levels and subclinical atherosclerotic markers in patients with CVD or hypertension, however, the studies in patients with T2DM are rare. Therefore, elucidating the effect of hemoglobin levels on the development of atherosclerosis in patients with diabetes mellitus has great clinical significance. In the PROLOGUE study population, 21.3% of participants had baseline anemia events. If patients with T2DM and anemia benefit from sitagliptin treatment, that is important from a public health standpoint, as its use could slow ICA-IMT progression in nearly one-fourth of the T2DM patients with anemia.

The etiology of anemia in patients with diabetes has been previously reported and is considered to be multifactorial. In addition to diabetic nephropathy, the inflammation, nutritional deficiency, and hormone changes are all causes of anemia. Renal tubules are the main sites for the production of erythropoietin [38]. Systemic inflammation, reduced red cell survival, functional erythropoietin deficiency, and erythropoietin resistance caused by renal tubular dysfunction in patients with diabetes may lead to insufficient erythropoietin response [31, 39, 40]. In addition, absolute or relative (functional) iron deficiency is also a major cause of anemia in diabetes. In general, relative iron deficiency is more common and is closely related to the upregulation of inflammatory cytokines and impaired tissue responsiveness to erythropoietin. Previous studies have shown a link between anemia and CVD in diabetic patients. Even mild anemia can lead to serious complications in CVD, such as myocardial infarction [41, 42]. According to physiological theories, anemia affects tissue perfusion and oxygenation by a reduction in the oxygen-carrying ability of blood due to decreases in hemoglobin concentration and the change of blood apparent viscosity resulting from low hematocrit [43]. And blood viscosity can affect systemic vascular resistance, which leads to cardiovascular hemodynamics changes. Moreover, anemia has been suggested to be directly involved in tissue protection and regulation of cardiovascular homeostasis by affecting the atypical functions of erythrocytes. Erythrocytes are an important part of blood antioxidant capacity. Anemia significantly weakened the antioxidant system [44]. The activation of erythropoiesis can lead to the decrease of oxidative stress, which in turn prevents atherosclerotic progression [45]. Atherosclerosis is one of the main factors contributing to an increase of CIMT [46]. Anemia may lead to endothelial dysfunction and decrease the level of nitric oxide (NO) by reducing endothelial shear stress, which in turn accelerates atherosclerosis by increasing the oxidation of low-density lipoprotein (LDL) [47–50]. Ganidagli et al. found that the patient (LH group) with the highest hemoglobin variability had the highest CIMT (LN: 0.601 mm, LH: 0.744 mm, NH: 0.604 mm, *p* < 0.001) [51]. Dogan and Citak found that low levels of hemoglobin were associated with increased CIMT in children with *β*-thalassemia major [52]. These findings support that changes in hemoglobin levels may lead to arterial remodeling. In the *post hoc* analysis, we also found that the baseline CIMT parameters of the anemic subgroup were higher than those of the nonanemic subgroup.

Our results suggest that patients with diabetes may be more significant to see the benefits of sitagliptin treatment before the development of anemia. For patients with anemia, it may be difficult to see the benefit of sitagliptin treatment. There is biologic plausibility of the findings that patients with T2DM and nonanemia can benefit from sitagliptin treatment. Anemia is associated with increased progression of atherosclerosis, increased carotid artery intimal-medial thickness, and cardiac hypertrophy [53]. The pathophysiological process of atherosclerosis in patients with anemia may be more advanced. These patients may not be able to benefit from sitagliptin treatment. In addition, the control of blood glucose helps to improve the tissue characteristics of carotid artery plaque. Although the decrease in HbA1c levels in the anemic subgroup was greater in the sitagliptin group than that in the conventional group, we consider that most patients have achieved relatively good blood glucose control and strict management of risk factors. Therefore, the progression of CIMT may be partially affected by anemia, although the additional effect of sitagliptin on the progression of CIMT was not shown in patients with risk factors for anemia. As an adjunct treatment for statins, angiotensin-converting enzyme inhibitors and angiotensin II receptor blockers, it seems to have a unique or additive antiatherosclerotic effects in T2DM patients without anemia. Previous studies have confirmed that statins and angiotensin-converting enzyme inhibitors can reduce the development of atherosclerosis in T2DM patients [54, 55]. The T2DM patients without anemia risk factors should choose more appropriate drugs for prevention and treatment. Furthermore, we speculate that a higher frequency of the use of angiotensin II receptor blocker and thiazolidinedione in anemia subgroup may be part of the reason for this result. Because the inhibitory effect of angiotensin II receptor blocker and thiazolidinedione on CIMT has also been confirmed [56, 57], this may mask or diminish the effectiveness of investigational drugs. In the PROLOGUE study, patients inevitably took other drugs, and it was difficult to rule out their effects. However, we reiterate that the real intention of the present study as an exploratory subgroup analysis is to explore whether sitagliptin can have an additional effect on the CIMT process. Our subgroup study did not find that sitagliptin slowed the progression of CIMT in patients with T2DM and anemia. The precise reasons are not clear. Tanaka et al. pointed out that T2DM patients with or without previous CV events were recruited in the PROLOGUE study [58], while other studies investigating the effects of DPP-4 inhibitors only recruited T2DM patients without events [24, 25].

Our study has the following limitations. First, it is a post hoc subanalysis using data obtained from the PROLOGUE study. The analysis was not part of the original statistical plan. Therefore, the result needs to be treated with caution. Second, other noninvestigational drugs such as antidiabetic, antihyperlipidemic, and antihypertensive may influence the CIMT progression, although the baseline drug therapies were almost matched in this post hoc analysis. Third, the sample size may not be sufficient to detect significant differences in CIMT variables between treatment groups in the anemia subgroup. In order to confirm our findings, it is necessary to design a prespecified study with a large sample size.

## 5. Conclusions

In this post hoc study of PROLOGUE study data, the patients with T2DM and nonanemia can obtain better antiatherosclerotic effects in the sitagliptin treatment group compared with the conventional group. Since this finding may be attributed to sampling variability, therefore, it would be premature to conclude that sitagliptin treatment significantly inhibited CIMT progression. Further research is needed.

## Figures and Tables

**Table 1:** Baseline clinical characteristics of the study population.

Variable	Anemia (NO, *n* = 343)		*P*	Anemia (YES, *n* = 94)		*P*
	Sitagliptin group (*n* = 175)	Conventional (*n* = 168)		Sitagliptin group (*n* = 44)	Conventional (*n* = 50)	
Mean age (years)	68.18 ± 8.87	68.16 ± 9.34	0.982	73.95 ± 8.27	73.56 ± 6.95	0.802
Gender (male), *n* (%)	118 (67.4%)	114 (67.9%)	0.932	25 (56.8%)	36 (72.0%)	0.124
BMI (kg/m^2^)	25.55 ± 4.14	25.25 ± 4.05	0.504	24.24 ± 3.83	23.75 ± 3.54	0.521
Dyslipidemia (%)	132 (75.4%)	117 (69.6%)	0.230	28 (63.6%)	30 (60.0%)	0.717
Cerebral infarction (%)	16 (9.1%)	17 (10.1%)	0.759	4 (9.1%)	8 (16.0%)	0.317
Myocardial infarction (%)	34 (19.4%)	38 (22.6%)	0.468	10 (22.7%)	17 (34.0%)	0.228
PCI (%)	42 (24.0%)	50 (29.8%)	0.229	16 (36.4%)	19 (38.0%)	0.870
CABG (%)	16 (9.1%)	11 (6.5%)	0.372	3 (6.8%)	5 (10.0%)	0.581
Chronic heart failure (%)	14 (8.0%)	16 (9.5%)	0.618	1 (2.3%)	10 (20.0%)	0.008
Arrhythmia (%)	28 (16.0%)	23 (13.7%)	0.548	4 (9.1%)	9 (18.0%)	0.212
Stroke (%)	21 (12.0%)	21 (12.5%)	0.888	5 (11.4%)	9 (18.0%)	0.367
SBP (mmHg)	130.19 ± 15.76	128.08 ± 15.73	0.215	130.18 ± 14.87	131.02 ± 19.10	0.812
DBP (mmHg)	73.50 ± 10.65	72.81 ± 11.20	0.560	70.36 ± 10.70	68.06 ± 11.80	0.327
HbA1c (%)	6.99 ± 0.69	6.96 ± 0.54	0.652	6.80 ± 0.36	6.95 ± 0.59	0.138
FPG (mg/dL)	140.15 ± 43.86	137.31 ± 35.10	0.520	130.52 ± 32.32	126.06 ± 42.18	0.579
Non-HDL cholesterol (mg/dL)	126.16 ± 29.02	125.99 ± 31.19	0.958	107.12 ± 28.32	103.34 ± 24.23	0.494
Serum creatinine (mg/dL)	0.83 ± 0.21	0.82 ± 0.22	0.937	0.97 ± 0.29	0.97 ± 0.27	0.997
eGFR (mL/min/1.73m^2^)	68.85 ± 16.66	69.64 ± 17.55	0.671	56.83 ± 17.57	58.58 ± 16.23	0.617
Mean CCA IMT (mm)	0.819 ± 0.146	0.816 ± 0.161	0.860	0.833 ± 0.222	0.904 ± 0.255	0.673
Mean bulb IMT (mm)	1.060 ± 0.381	1.129 ± 0.451	0.182	1.330 ± 0.550	1.173 ± 0.352	0.154
Mean ICA IMT (mm)	0.782 ± 0.279	0.772 ± 0.319	0.780	0.799 ± 0.246	0.816 ± 0.268	0.793
Max CCA IMT (mm)	1.037 ± 0.197	1.047 ± 0.215	0.659	1.132 ± 0.316	1.185 ± 0.370	0.463
Max bulb IMT (mm)	1.522 ± 0.570	1.603 ± 0.677	0.286	1.865 ± 0.843	1.741 ± 0.537	0.459
Max ICA IMT (mm)	1.059 ± 0.375	1.039 ± 0.440	0.700	1.057 ± 0.333	1.135 ± 0.397	0.399
Plaque area (mm^2^)	11.083 ± 7.311	11.283 ± 6.656	0.837	12.903 ± 7.992	13.085 ± 14.233	0.955
Plaque gray scale median	52.335 ± 23.765	51.094 ± 18.747	0.675	44.525 ± 15.908	55.956 ± 28.407	0.077

Abbreviation: BMI: body mass index; PCI: percutaneous coronary intervention; CABG: coronary artery bypass grafting; SBP: systolic blood pressure; DBP: diastolic blood pressure; FPG: fasting plasma glucose; HDL: high-density lipoprotein; eGFR: estimated glomerular filtration rate; CCA: common carotid artery; IMT: intima-media thickness; ICA: internal carotid artery.

**Table 2:** Baseline-adjusted mean and group difference between treatment groups.

Variable	Time point	Anemia (NO, *n* = 343)			Anemia (YES, *n* = 94)			
		Baseline-adjusted mean ± SE		Group difference in baseline-adjusted mean (95% CI)	*P*	Baseline-adjusted mean ± SE		Group difference in baseline-adjusted mean (95% CI)	*P*
		Sitagliptin group (*n* = 175)	Conventional (*n* = 168)			Sitagliptin group (*n* = 44)	Conventional (*n* = 50)		
BMI (kg/m^2^)	12 mo	25.289 ± 0.099	25.154 ± 0.101	0.135 (−0.118 to 0.388)	0.294	24.104 ± 0.191	24.294 ± 0.193	−0.190 (−0.669 to 0.290)	0.433
	24 mo	25.112 ± 0.124	25.382 ± 0.128	−0.270 (−0.585 to 0.046)	0.094	24.154 ± 0.220	24.281 ± 0.222	−0.127 (−0.693 to 0.439)	0.655

SBP (mmHg)	12 mo	128.561 ± 1.221	130.439 ± 1.238	−1.878 (−4.968 to 1.213)	0.233	129.517 ± 2.754	128.137 ± 2.677	1.381 (−5.621 to 8.382)	0.695
	24 mo	129.209 ± 1.392	129.383 ± 1.385	−0.174 (−3.679 to 3.331)	0.922	132.486 ± 2.862	131.188 ± 2.698	1.298 (−5.787 to 8.382)	0.716

DBP (mmHg)	12 mo	72.438 ± 0.879	74.408 ± 0.894	−1.970 (−4.199 to 0.260)	0.083	68.025 ± 1.673	69.585 ± 1.641	−1.560 (−5.882 to 2.762)	0.474
	24 mo	72.801 ± 0.905	72.817 ± 0.904	−0.016 (−2.302 to 2.269)	0.989	72.786 ± 1.808	69.435 ± 1.723	3.351 (−1.216 to 7.918)	0.148

HbA1c (%)	12 mo	6.604 ± 0.049	6.711 ± 0.049	−0.107 (−0.230 to 0.017)	0.090	6.386 ± 0.069	6.471 ± 0.067	−0.085 (−0.260 to 0.090)	0.337
	24 mo	6.575 ± 0.054	6.721 ± 0.054	−0.146 (−0.282 to −0.010)	0.035	6.513 ± 0.112	6.687 ± 0.103	−0.175 (−0.450 to 0.101)	0.210

FPG (mg/dL)	12 mo	132.904 ± 3.020	131.049 ± 2.945	1.854 (−5.590 to 9.299)	0.624	122.157 ± 5.343	118.647 ± 5.152	3.510 (−9.977 to 16.998)	0.605
	24 mo	129.971 ± 3.083	130.994 ± 2.994	−1.023 (−8.747 to 6.702)	0.795	118.844 ± 5.938	117.265 ± 5.689	1.579 (−13.422 to 16.580)	0.834

Non-HDL cholesterol (mg/dL)	12 mo	124.406 ± 2.061	127.221 ± 2.065	−2.814 (−7.969 to 2.341)	0.284	110.024 ± 3.506	105.687 ± 3.552	4.337 (−4.462 to 13.136)	0.329
	24 mo	125.432 ± 2.193	126.784 ± 2.223	−1.353 (−6.914 to 4.209)	0.632	101.153 ± 4.287	103.960 ± 3.994	−2.806 (−13.142 to 7.529)	0.589

Serum creatinine (mg/dL)	12 mo	0.840 ± 0.009	0.826 ± 0.009	0.014 (−0.008 to 0.035)	0.212	0.981 ± 0.023	0.975 ± 0.023	0.006 (−0.052 to 0.065)	0.828
	24 mo	0.861 ± 0.014	0.850 ± 0.014	0.011 (−0.023 to 0.045)	0.540	1.002 ± 0.038	0.999 ± 0.035	0.004 (−0.090 to 0.097)	0.938

eGFR (mL/min/1.73m^2^)	12 mo	67.663 ± 0.770	68.850 ± 0.787	−1.187 (−3.143 to 0.768)	0.233	57.007 ± 1.224	57.781 ± 1.207	−0.774 (−3.899 to 2.350)	0.623
	24 mo	66.450 ± 0.838	66.696 ± 0.847	−0.247 (−2.375 to 1.881)	0.820	55.854 ± 1.946	57.659 ± 1.797	−1.805 (−6.585 to 2.975)	0.453

Mean CCA IMT (mm)	12 mo	0.812 ± 0.008	0.808 ± 0.008	0.005 (−0.015 to 0.024)	0.655	0.877 ± 0.018	0.898 ± 0.019	−0.021 (−0.066 to 0.024)	0.349
	24 mo	0.819 ± 0.007	0.823 ± 0.007	−0.003 (−0.022 to 0.015)	0.718	0.894 ± 0.015	0.900 ± 0.015	−0.007 (−0.043 to 0.030)	0.724

Mean bulb IMT (mm)	12 mo	1.145 ± 0.037	1.171 ± 0.037	−0.026 (−0.118 to 0.066)	0.583	1.299 ± 0.079	1.221 ± 0.091	0.078 (−0.113 to 0.270)	0.412
	24 mo	1.164 ± 0.036	1.167 ± 0.036	−0.003 (−0.093 to 0.087)	0.945	1.308 ± 0.070	1.226 ± 0.068	0.082 (−0.088 to 0.252)	0.335

Mean ICA IMT (mm)	12 mo	0.908 ± 0.041	0.931 ± 0.040	−0.023 (−0.126 to 0.081)	0.666	0.909 ± 0.099	0.840 ± 0.105	0.068 (−0.165 to 0.302)	0.557
	24 mo	0.740 ± 0.031	0.844 ± 0.030	−0.104 (−0.182 to −0.026)	0.009	0.878 ± 0.058	0.814 ± 0.051	0.064 (−0.069 to 0.196)	0.337

Max CCA IMT (mm)	12 mo	1.035 ± 0.013	1.017 ± 0.013	0.018 (−0.014 to 0.049)	0.278	1.157 ± 0.027	1.154 ± 0.029	0.003 (−0.065 to 0.071)	0.937
	24 mo	1.046 ± 0.013	1.042 ± 0.013	0.004 (−0.029 to 0.036)	0.816	1.182 ± 0.024	1.145 ± 0.023	0.037 (−0.022 to 0.096)	0.217

Max bulb IMT (mm)	12 mo	1.560 ± 0.051	1.606 ± 0.051	−0.046 (−0.171 to 0.080)	0.473	1.704 ± 0.108	1.516 ± 0.121	0.188 (−0.068 to 0.445)	0.146
	24 mo	1.656 ± 0.049	1.656 ± 0.049	0.000 (−0.122 to 0.121)	0.996	1.831 ± 0.097	1.664 ± 0.093	0.167 (−0.062 to 0.396)	0.149

Max ICA IMT (mm)	12 mo	1.207 ± 0.054	1.223 ± 0.052	−0.016 (−0.149 to 0.118)	0.816	1.232 ± 0.131	1.165 ± 0.136	0.067 (−0.239 to 0.373)	0.659
	24 mo	1.012 ± 0.043	1.154 ± 0.043	−0.142 (−0.252 to −0.033)	0.011	1.176 ± 0.090	1.129 ± 0.077	0.047 (−0.155 to 0.249)	0.641

Plaque area (mm^2^)	12 mo	12.141 ± 0.810	12.504 ± 0.678	−0.363 (−2.271 to 1.545)	0.707	17.922 ± 3.381	14.411 ± 4.695	3.511 (−2.935 to 9.957)	0.267
	24 mo	11.743 ± 0.619	10.568 ± 0.569	1.175 (−0.334 to 2.684)	0.126	13.401 ± 1.739	11.910 ± 1.452	1.491 (−2.062 to 5.045)	0.401

Plaque gray scale median	12 mo	60.939 ± 5.327	62.247 ± 4.424	−1.308 (−13.856 to 11.239)	0.837	57.477 ± 8.156	55.640 ± 11.599	1.837 (−13.740 to 17.415)	0.807
	24 mo	49.202 ± 2.738	54.482 ± 2.518	−5.280 (−11.967 to 1.407)	0.121	49.298 ± 5.505	48.248 ± 4.532	1.050 (−10.193 to 12.293)	0.851

Abbreviation: BMI: body mass index; SBP: systolic blood pressure; DBP: diastolic blood pressure; FPG: fasting plasma glucose; CCA: common carotid artery; IMT: intima-media thickness; ICA: internal carotid artery.

**Table 3:** Changes in baseline-adjusted mean and group difference between treatment groups.

Variable	Time point	Anemia (NO, *n* = 343)				Anemia (YES, *n* = 94)			
		Baseline-adjusted mean ± SE		Group difference in baseline-adjusted mean (95% CI)	*P*	Baseline-adjusted mean ± SE		Group difference in baseline-adjusted mean (95% CI)	*P*
		Sitagliptin group (*n* = 175)	Conventional (*n* = 168)			Sitagliptin group (*n* = 44)	Conventional (*n* = 50)		
BMI (kg/m^2^)	12 mo	−0.019 ± 0.099	−0.154 ± 0.101	0.135 (−0.118 to 0.388)	0.294	−0.102 ± 0.191	0.087 ± 0.193	−0.190 (−0.669 to 0.290)	0.433
	24 mo	−0.381 ± 0.124	−0.112 ± 0.128	−0.270 (−0.585 to 0.046)	0.094	−0.129 ± 0.220	−0.001 ± 0.222	−0.127 (−0.693 to 0.439)	0.655

SBP (mmHg)	12 mo	−0.757 ± 1.221	1.121 ± 1.238	−1.878 (−4.968 to 1.213)	0.233	−1.483 ± 2.754	−2.863 ± 2.677	1.381 (−5.621 to 8.382)	0.695
	24 mo	0.142 ± 1.392	0.316 ± 1.385	−0.174 (−3.679 to 3.331)	0.922	1.730 ± 2.862	0.432 ± 2.698	1.298 (−5.787 to 8.382)	0.716

DBP (mmHg)	12 mo	−0.821 ± 0.879	1.148 ± 0.894	−1.970 (−4.199 to 0.260)	0.083	−2.062 ± 1.673	−0.502 ± 1.641	−1.560 (−5.882 to 2.762)	0.474
	24 mo	−0.230 ± 0.905	−0.213 ± 0.904	−0.016 (−2.302 to 2.269)	0.989	2.965 ± 1.808	−0.386 ± 1.723	3.351 (−1.216 to 7.918)	0.148

HbA1c (%)	12 mo	−0.364 ± 0.049	−0.258 ± 0.049	−0.107 (−0.230 to 0.017)	0.090	−0.498 ± 0.069	−0.413 ± 0.067	−0.085 (−0.260 to 0.090)	0.337
	24 mo	−0.363 ± 0.054	−0.217 ± 0.054	−0.146 (−0.282 to −0.010)	0.035	−0.390 ± 0.112	−0.215 ± 0.103	−0.175 (−0.450 to 0.101)	0.210

FPG (mg/dL)	12 mo	−5.041 ± 3.020	−6.896 ± 2.945	1.854 (−5.590 to 9.299)	0.624	−4.451 ± 5.343	−7.961 ± 5.152	3.510 (−9.977 to 16.998)	0.605
	24 mo	−6.306 ± 3.083	−5.283 ± 2.994	−1.023 (−8.747 to 6.702)	0.795	−7.595 ± 5.938	−9.174 ± 5.689	1.579 (−13.422 to 16.580)	0.834

Non-HDL cholesterol (mg/dL)	12 mo	−2.700 ± 2.061	0.114 ± 2.065	−2.814 (−7.969 to 2.341)	0.284	5.642 ± 3.506	1.305 ± 3.552	4.337 (−4.462 to 13.136)	0.329
	24 mo	−1.207 ± 2.193	0.146 ± 2.223	−1.353 (−6.914 to 4.209)	0.632	−3.986 ± 4.287	−1.179 ± 3.994	−2.806 (−13.142 to 7.529)	0.589

Serum creatinine (mg/dL)	12 mo	0.020 ± 0.009	0.007 ± 0.009	0.014 (−0.008 to 0.035)	0.212	0.010 ± 0.023	0.003 ± 0.023	0.006 (−0.052 to 0.065)	0.828
	24 mo	0.047 ± 0.014	0.036 ± 0.014	0.011 (−0.023 to 0.045)	0.540	0.026 ± 0.038	0.023 ± 0.035	0.004 (−0.090 to 0.097)	0.938

eGFR (mL/min/1.73m^2^)	12 mo	−1.967 ± 0.770	−0.779 ± 0.787	−1.187 (−3.143 to 0.768)	0.233	−1.040 ± 1.224	−0.265 ± 1.207	−0.774 (−3.899 to 2.350)	0.623
	24 mo	−3.637 ± 0.838	−3.391 ± 0.847	−0.247 (−2.375 to 1.881)	0.820	−1.934 ± 1.946	−0.128 ± 1.797	−1.805 (−6.585 to 2.975)	0.453

Mean CCA IMT (mm)	12 mo	0.001 ± 0.008	−0.004 ± 0.008	0.005 (−0.015 to 0.024)	0.655	−0.022 ± 0.018	−0.001 ± 0.019	−0.021 (−0.066 to 0.024)	0.349
	24 mo	0.008 ± 0.007	0.011 ± 0.007	−0.003 (−0.022 to 0.015)	0.718	−0.013 ± 0.015	−0.007 ± 0.015	−0.007 (−0.043 to 0.030)	0.724

Mean bulb IMT (mm)	12 mo	0.056 ± 0.037	0.082 ± 0.037	−0.026 (−0.118 to 0.066)	0.583	0.057 ± 0.079	−0.021 ± 0.091	0.078 (−0.113 to 0.270)	0.412
	24 mo	0.060 ± 0.036	0.063 ± 0.036	−0.003 (−0.093 to 0.087)	0.945	0.056 ± 0.070	−0.026 ± 0.068	0.082 (−0.088 to 0.252)	0.335

Mean ICA IMT (mm)	12 mo	0.112 ± 0.041	0.134 ± 0.040	−0.023 (−0.126 to 0.081)	0.666	0.078 ± 0.099	0.010 ± 0.105	0.068 (−0.165 to 0.302)	0.557
	24 mo	−0.024 ± 0.031	0.079 ± 0.030	−0.104 (−0.182 to −0.026)	0.009	0.062 ± 0.058	−0.002 ± 0.051	0.064 (−0.069 to 0.196)	0.337

Max CCA IMT (mm)	12 mo	0.002 ± 0.013	−0.015 ± 0.013	0.018 (−0.014 to 0.049)	0.278	0.000 ± 0.027	−0.003 ± 0.029	0.003 (−0.065 to 0.071)	0.937
	24 mo	0.015 ± 0.013	0.011 ± 0.013	0.004 (−0.029 to 0.036)	0.816	0.015 ± 0.024	−0.022 ± 0.023	0.037 (−0.022 to 0.096)	0.217

Max bulb IMT (mm)	12 mo	0.006 ± 0.051	0.051 ± 0.051	−0.046 (−0.171 to 0.080)	0.473	−0.058 ± 0.108	−0.246 ± 0.121	0.188 (−0.068 to 0.445)	0.146
	24 mo	0.079 ± 0.049	0.080 ± 0.049	0.000 (−0.122 to 0.121)	0.996	0.059 ± 0.097	−0.108 ± 0.093	0.167 (−0.062 to 0.396)	0.149

Max ICA IMT (mm)	12 mo	0.121 ± 0.054	0.137 ± 0.052	−0.016 (−0.149 to 0.118)	0.816	0.101 ± 0.131	0.034 ± 0.136	0.067 (−0.239 to 0.373)	0.659
	24 mo	−0.019 ± 0.043	0.123 ± 0.043	−0.142 (−0.252 to −0.033)	0.011	0.073 ± 0.090	0.026 ± 0.077	0.047 (−0.155 to 0.249)	0.641

Plaque area (mm^2^)	12 mo	0.281 ± 0.810	0.644 ± 0.678	−0.363 (−2.271 to 1.545)	0.707	3.158 ± 3.381	−0.353 ± 4.695	3.511 (−2.935 to 9.957)	0.267
	24 mo	0.814 ± 0.619	−0.361 ± 0.569	1.175 (−0.334 to 2.684)	0.126	−0.840 ± 1.739	−2.331 ± 1.452	1.491 (−2.062 to 5.045)	0.401

Plaque gray scale median	12 mo	8.507 ± 5.327	9.815 ± 4.424	−1.308 (−13.856 to 11.239)	0.837	10.639 ± 8.156	8.802 ± 11.599	1.837 (−13.740 to 17.415)	0.807
	24 mo	−1.662 ± 2.738	3.617 ± 2.518	−5.280 (−11.967 to 1.407)	0.121	−1.755 ± 5.505	−2.805 ± 4.532	1.050 (−10.193 to 12.293)	0.851

Abbreviation: BMI: body mass index; SBP: systolic blood pressure; DBP: diastolic blood pressure; FPG: fasting plasma glucose; CCA: common carotid artery; IMT: intima-media thickness; ICA: internal carotid artery.

**Table 4:** Frequency of the use of antidiabetic and other agents.

Variable	Time point	Anemia (NO)			Anemia (YES)		
		Sitagliptin group	Conventional	*P*	Sitagliptin group	Conventional	*P*
Sulfonylurea	Baseline	50 (28.6%)	42 (25.0%)	0.455	5 (11.4%)	10 (20.0%)	0.254
	12 mo	35 (21.3%)	55 (35.3%)	0.006	4 (10.8%)	11 (25.0%)	0.102
	24 mo	32 (20.8%)	51 (34.2%)	0.009	3 (8.6%)	10 (23.3%)	0.083

Metformin	Baseline	24 (13.7%)	19 (11.3%)	0.501	8 (18.2%)	13 (26.0%)	0.364
	12 mo	30 (18.3%)	50 (32.1%)	0.004	8 (21.6%)	18 (40.9%)	0.064
	24 mo	34 (22.1%)	50 (33.6%)	0.026	9 (25.7%)	18 (41.9%)	0.136

*α*-Glucosidase inhibitor	Baseline	57 (32.6%)	46 (27.4%)	0.294	14 (31.8%)	20 (40.0%)	0.410
	12 mo	42 (25.6%)	61 (39.1%)	0.010	11 (29.7%)	23 (52.3%)	0.041
	24 mo	35 (22.7%)	58 (38.9%)	0.002	10 (28.6%)	23 (53.5%)	0.027

Thiazolidinedione	Baseline	35 (20.0%)	36 (21.4%)	0.744	17 (38.6%)	17 (34.0%)	0.641
	12 mo	25 (15.2%)	45 (28.8%)	0.003	13 (35.1%)	19 (43.2%)	0.461
	24 mo	24 (15.6%)	47 (31.5%)	0.001	11 (31.4%)	15 (34.9%)	0.747

Glinide	Baseline	5 (2.9%)	15 (8.9%)	0.016	2 (4.5%)	4 (8.0%)	0.494
	12 mo	1 (0.6%)	21 (13.5%)	0.0001	3 (8.1%)	4 (9.1%)	0.875
	24 mo	1 (0.6%)	17 (11.4%)	0.0001	2 (5.7%)	4 (9.3%)	0.554

Statin	Baseline	133 (76.0%)	125 (74.4%)	0.732	33 (75.0%)	36 (72.0%)	0.743
	12 mo	122 (74.4%)	113 (72.4%)	0.692	26 (70.3%)	30 (68.2%)	0.839
	24 mo	114 (74.0%)	106 (71.1%)	0.573	25 (71.4%)	29 (67.4%)	0.704

Fibrate	Baseline	1 (0.6%)	3 (1.8%)	0.295	1 (2.3%)	0 (0.0%)	0.284
	12 mo	1 (0.6%)	3 (1.9%)	0.291	1 (2.7%)	0 (0.0%)	0.273
	24 mo	1 (0.6%)	3 (2.0%)	0.298	1 (2.9%)	0 (0.0%)	0.265

Angiotensin II receptor blocker	Baseline	98 (56.0%)	84 (50.0%)	0.266	31 (70.5%)	27 (54.0%)	0.102
	12 mo	90 (54.9%)	80 (51.3%)	0.519	27 (73.0%)	24 (54.5%)	0.087
	24 mo	87 (56.5%)	76 (51.0%)	0.338	27 (77.1%)	23 (53.5%)	0.030

Angiotensin-converting enzyme inhibitor	Baseline	20 (11.4%)	25 (14.9%)	0.344	6 (13.6%)	11 (22.0%)	0.293
	12 mo	20 (12.2%)	23 (14.7%)	0.504	3 (8.1%)	9 (20.5%)	0.119
	24 mo	16 (10.4%)	21 (14.1%)	0.325	3 (8.6%)	10 (23.3%)	0.083

Number of patients in the NO anemia subgroup = baseline: 175 (Sita), 168 (Con); 12 months: 164 (Sita), 156 (Con); 24 months: 154 (Sita), 149 (Con). Number of patients in the anemia subgroup = baseline: 44 (Sita), 50 (Con); 12 months: 37 (Sita), 44 (Con); 24 months: 35 (Sita), 43 (Con). Data are expressed as number (%).

## Data Availability

Data and materials are available upon the Dryad Digital Repository. (https://doi.org/10.5061/dryad.qt743).
